# Low Levels of Adropin Predict Adverse Clinical Outcomes in Outpatients with Newly Diagnosed Prediabetes after Acute Myocardial Infarction

**DOI:** 10.3390/biomedicines12081857

**Published:** 2024-08-15

**Authors:** Tetiana A. Berezina, Oleksandr O. Berezin, Uta C. Hoppe, Michael Lichtenauer, Alexander E. Berezin

**Affiliations:** 1 Department of Internal Medicine and Nephrology, VitaCenter, 69000 Zaporozhye, Ukraine; talexberezina@gmail.com; 2 Department of Alter Psychiatrie, Luzerner Psychiatrie AG, 4915 St. Urban, Switzerland; oleksandr.berezin@lups.ch; 3 Department of Internal Medicine II, Division of Cardiology, Paracelsus Medical University, 5020 Salzburg, Austria; u.hoppe@salk.at (U.C.H.); m.lichtenauer@salk.at (M.L.)

**Keywords:** prediabetes, adropin, acute myocardial infarction, heart failure, cardiovascular death, all-cause mortality, prognosis

## Abstract

Adropin—a multifunctional peptide with tissue-protective capacity that regulates energy homeostasis, sensitivity to insulin and inflammatory response—seems to show an inverse association with the presence of cardiovascular and renal diseases, obesity and diabetes mellitus in the general population. The purpose of the study is to elucidate whether adropin may be a plausible predictive biomarker for clinical outcomes in post-ST elevation of myocardial infarction (STEMI) patients with newly diagnosed prediabetes according to the American Diabetes Association criteria. A total of 1214 post-STEMI patients who received percutaneous coronary intervention were identified in a local database of the private hospital “Vita Center” (Zaporozhye, Ukraine). Between November 2020 and June 2024, we prospectively enrolled 498 patients with prediabetes in this open prospective cohort study and followed them for 3 years. The combined clinical endpoint at follow-up was defined as cardiovascular death due to acute myocardial infarction, heart failure, sudden death due to arrhythmia or cardiac surgery, and/or all-cause death. We identified 126 clinical events and found that serum levels of adropin < 2.15 ng/mL (area under the curve = 0.836; 95% confidence interval = 0.745–0.928; sensitivity = 84.9%; specificity = 72.7%; likelihood ratio = 3.11; *p* = 0.0001) predicted clinical outcomes. Multivariate logistic regression showed that a Gensini score ≥ 32 (Odds ratio [OR] = 1.07; *p* = 0.001), adropin ≤ 2.15 ng/mL (OR = 1.18; *p* = 0.001), use of SGLT2i (OR = 0.94; *p* = 0.010) and GLP-1 receptor agonist (OR = 0.95; *p* = 0.040) were independent predictors of clinical outcome. Kaplan–Meier plots showed that patients with lower adropin levels (≤2.15 ng/mL) had worse clinical outcomes compared to patients with higher adropin levels (>2.15 ng/mL). In conclusion, low levels of adropin (≤2.15 ng/mL) independently predicted clinical outcomes in post-STEMI patients with newly detected prediabetes and improved the discriminative ability of the Gensini score for 3-year follow-up events. Future clinical studies are needed to clarify whether adropin is a promising molecule to be incorporated into conventional risk scores for the prediction of MACCEs after STEMI.

## 1. Introduction

Prediabetes, as an intermediate stage between normal glucose homeostasis and established diabetes mellitus (DM), is a common non-cardiovascular outcome after ST elevation of myocardial infarction (STEMI) [1]. Although the global prevalence of prediabetes is approximately 22% (ranging from 9.6% to 37.2%), between 35% and 68% of STEMI patients with no prior diagnosis of DM at discharge from the hospital demonstrate newly detected fasting glucose and/or abnormal glucose tolerance [2,3,4].

Generally, prediabetes is defined as a metabolic condition that is associated with hyperglycemia in the range of 5.6–6.9 mmol/L or glycated hemoglobin (HbA1c) in the range of 5.7–6.4%, which are typically higher than normal ranges but lower than the conventional thresholds for diabetes mellitus [2]. Although the diagnostic criteria for the condition are variable and differ between some clinical guidelines, early diagnosis and thorough screening of prediabetes are considered essential steps not only to prevent diabetes mellitus but also to predict cardiovascular complications [5,6]. Indeed, the concept of “prediabetes” is based on a predictive model that utilized the high composite risk of developing diabetes mellitus and CVD, including acute myocardial infarction, heart failure and asymptomatic atherosclerosis, by potentiating the effects of metabolic abnormalities, such as hyperglycemia, lipid toxicity, insulin resistance and concomitant metabolic condition, including abdominal obesity [2].

Although prediabetes was not found to be a strong predictor of 30-day outcome in STEMI patients without elevated glycosylated hemoglobin (HbA1c) levels on admission (5.7–6.4%), diagnosed prediabetes or DM was independently associated with long-term all-cause and cardiovascular (CV) mortality and hospital readmission [7,8,9,10]. In addition, prediabetes sufficiently increases the risk of complications after STEMI and percutaneous coronary intervention (PCI), including heart failure (HF) and sudden death [11,12,13,14].

The main underlying pathogenic mechanisms by which newly diagnosed prediabetes among non-DM individuals after STEMI intervenes in CV outcomes may include (a) support of angiopathy through low-grade inflammation, oxidative stress, endothelial dysfunction, (b) adverse cardiac remodeling through accumulation of extracellular matrix, acceleration of coronary atherosclerosis, myocardial ischemia/necrosis, neurohumoral activation, (c) attenuation of endogenous reparative potency via genetic/epigenetic regulation of metabolic memory, insulin resistance, lipid toxicity, alteration of mitochondrial structure and function, impairment of cellular energy homeostasis, (d) worsening auto-paracrine cardiac function regulation, (e) potentiating the impact of conventional CV and non-CV risk factors, such as abdominal obesity, hypertension, dyslipidemia, chronic kidney disease, on target organs, PCI efficacy and long-term survival, etc. [15,16,17,18,19,20,21]. Overall, prediabetes rather reflects the continuum of the risk of microvascular and macrovascular outcomes than the stable abnormality of glucose metabolism. Indeed, individuals with known prediabetes had a higher prevalence of adnominal obesity and dyslipidemia with a more atherogenic lipid profile and an elevated risk of atherosclerotic cardiovascular disease. In this context, it should be noted that the multivariate impact of prediabetes on the clinical outcomes of patients after STEMI requires a rational approach to risk stratification.

Previous clinical studies have shown that the conventional scores, clinical signs/symptoms, echocardiographic features (left ventricular ejection fraction and global longitudinal strain) and biomarker models, including natriuretic peptides, cardiac troponins, HbA1c and C-reactive protein, had limited discriminative potency for long-term outcomes in stable prediabetes patients after STEMI effectively treated with PCI [22]. Indeed, only high concentrations of N-terminal brain natriuretic pro-peptide (NT-proBNP) and high-sensitivity troponin T (hs-TnT) predicted incident CV events in patients with prediabetes [23]. Of note, at least 50% of HF individuals, regardless of other CV risk factors, including prediabetes, diabetes, obesity and metabolic syndrome, exhibited near normal concentrations of NT-proBNP [24,25]. On the other hand, it has been established that the combination of echocardiographic parameters and circulating biomarkers along with traditional CV risk factors in individuals who are normoglycemic or with prediabetes may improve the risk prediction for both incident HF and total CV events in asymptomatic/stable CV disease [26,27]. It remains unclear what biomarker models are optimal for patients after STEMI with newly diagnosed prediabetes to be stratified at risk of further outcomes.

Adropin, a recently identified multifunctional pro-angiogenic neuroendocrine protein that belongs to a group of hepatokines, plays a crucial role in maintaining glucose and lipid homeostasis, regulation of insulin sensitivity and body weight, recruitment of endothelial progenitor cells, immune response, organ protection and attenuation of inflammation [28]. It is mainly produced by hepatocytes and has also been found in the brain, gastrointestinal tract and myocardium [28]. Adropin acts through direct interaction with the G-protein-coupled receptor and activates the NB-3/Notch and vascular endothelial growth factor (VEGF) receptor (VEGFR2)-PI3K-Akt and VEGFR2-Erk1/2 signaling pathways [29]. In STEMI, adropin was able to promote myocardial ischemia, prevent myocardial necrosis, neutrophil/macrophage infiltration of the necrotic area, modulate endothelial function and exert cardioprotective effects [30,31,32].

Adropin was found to be inversely associated with insulin resistance and body mass index [33]. On the other hand, adropin levels were often elevated in chronic HF patients, whereas low adropin levels were found in acute HF due to STEMI [34,35]. However, adropin levels in HF patients appear to be closely related to metabolic comorbidities and chronic kidney disease [36]. Overall, low levels of adropin have been associated with CV complications. In addition, low levels of adropin have been proposed as a novel biomarker for HF, renal outcomes, type 2 DM-induced adverse cardiac remodeling, atherosclerosis and cardiac cachexia [37,38,39]. Therefore, the measurement of adropin is promising in the context of investigating the impact of prediabetes on clinical outcomes after STEMI. The aim of this study is to determine whether adropin is a plausible predictive biomarker for clinical outcomes in post-STEMI patients with newly diagnosed prediabetes.

## 2. Materials and Methods

### 2.1. Patient Population and Study Design

**Figure 1:**
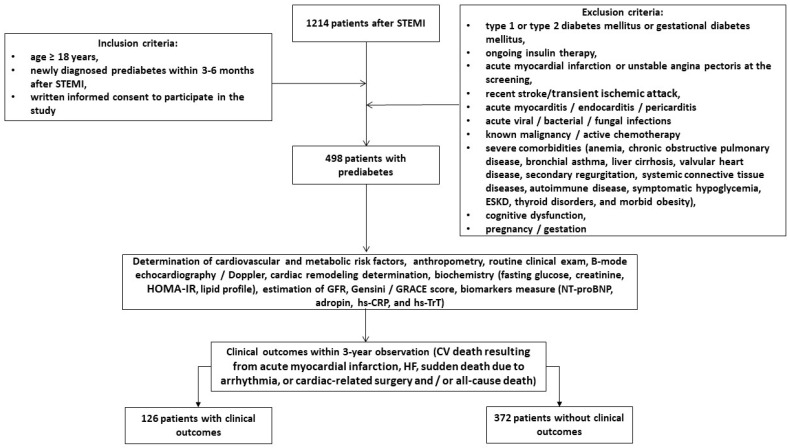
Flow chart of study design. Abbreviations: CV, cardiovascular; GRACE, Global Registry of Acute Coronary Events; HOMA-IR, homeostatic assessment model of insulin resistance; HF, heart failure; NT-proBNP, N-terminal brain natriuretic pro-peptide; hs-CRP, high-sensitivity C-reactive peptide; GFR, glomerular filtration rate; hs-TrT, high-sensitivity troponin T; STEMI, ST elevation of myocardial infarction; ESKD, end-stage kidney disease.

A total of 1214 post-STEMI patients who received PCI were identified in a local database of the private hospital “Vita Center” (Zaporozhye, Ukraine). Using the inclusion criteria (male and female aged ≥18 years, newly diagnosed prediabetes within 3–6 months after STEMI treated with successful PCI (TIMI score = 2–3), informed consent to participate in the study), we prospectively enrolled 498 patients with prediabetes in this open prospective cohort study and followed them for 3 years. The inclusion and exclusion criteria, as well as study procedures and determination of clinical outcomes, are outlined in Figure 1. The study was conducted between November 2020 and June 2024.

### 2.2. Demographics and Anthropomorphic Data Collection

Demographics and anthropomorphic data, basic clinical characteristics and comorbidities were collected at baseline and at the end of the study.

### 2.3. Clinical Outcomes Determination

The combined clinical endpoint of follow-up was defined as CV death resulting from acute myocardial infarction, HF, sudden death due to arrhythmia, or cardiac-related surgery and/or all-cause death.

### 2.4. Relevant Medical Data Collection

Over the three-year study period, we collected data on patients from a variety of sources, including medical records, databases, discharge summaries, autopsy reports and direct calls to patients, their relatives and/or their doctors. 

### 2.5. Determination of Prediabetes/Diabetes and Other Comorbidities

The identification of individuals at risk of prediabetes and type 2 diabetes was performed using the American Diabetes Association (ADA) Diabetes Risk Test [40]. We used the ADA criteria for prediabetes: glycosylated hemoglobin (HbA1c) = 5.7–6.4%, fasting plasma glucose = 5.6–6.9 mmol/L, 2-h plasma glucose during a 75 g oral glucose tolerance test (OGTT) = 7.8–11.0 mmol/L. 

Chronic kidney disease (CKD) [41], heart failure [42], hypertension [43] and dyslipidemia [44] were assessed according to current clinical guidelines.

### 2.6. Echocardiography Examination

All patients underwent echocardiographic and Doppler examinations performed by two blinded, highly experienced echocardiographers according to the guidelines of the American Society of Echocardiography [45]. The standard apical 2- and 4-chamber views were acquired at baseline and at the end of the study using a GE Healthcare Vivid E95 scanner (General Electric Company, Horton, Norway). The conventional hemodynamic parameters included left ventricular ejection fraction (LVEF) using Simpson’s method, left ventricular end-diastolic (LVEDV) and end-systolic (LVESV) volumes, left atrial volume index (LAVI), early diastolic blood filling (E) and mean longitudinal strain ratio (e’). The estimated E/e’ ratio was expressed as the ratio of the E wave velocity to the averaged medial and lateral e’ velocities. Left ventricular hypertrophy was defined as a left ventricular mass index (LVMI) ≥ 95 g/m^2^ in women or ≥115 g/m^2^ in men. Left ventricular global longitudinal strain (GLS) was obtained via 2D speckle tracking imaging after obtaining high-quality echocardiographic recordings during at least three cardiac cycles. Data were stored in DICOM format for further analysis.

### 2.7. Glomerular Filtration Rate and Insulin Resistance Determination

The conventional CKD-EPI formula was used to estimate the glomerular filtration rate (eGFR) [46]. The homeostatic assessment model of insulin resistance (HOMA-IR) was used to assess insulin resistance [47].

### 2.8. Post-STEMI Risk Determination and Assessment of Atherosclerosis Severity

All enrolled patients underwent coronary angiography, and the results were assessed by at least two interventional physicians. The severity of atherosclerosis was assessed using the Gensini score system [48]. The post-discharge Global Registry of Acute Coronary Events (GRACE) score was calculated for all patients with a discriminative survival of 3 years [49].

### 2.9. Blood Sampling 

Venous blood samples (3–5 mL) were collected from fasting patients in Vacutainer tubes at three time points: baseline and end of study. Pooled samples were centrifuged (3000 r/min, 30 min). Sera were collected and immediately frozen and stored at −70 °C until analysis before utilization. All routine biochemical tests were performed using standard biochemical techniques on a Roche P800 analyzer (Basel, Switzerland).

### 2.10. Biomarker Analysis

NT-proBNP, adropin, hs-TrT and hs-CRP were quantified in the serum using commercial enzyme-linked immunosorbent assay (ELISA) kits manufactured by Elabscience (Houston, TX, USA). The intra- and inter-assay coefficients of variations for all kits were <10%.

### 2.11. Statistics

The Kolmogorov–Smirnov test was used to assess normality and the Levene test to assess homogeneity. Continuous variables were presented as mean (M) and standard deviation (SD) or median (Me) and 25–75% interquartile range (IQR), depending on their distribution. Categorical variables were presented as proportions and percentages of the total. Chi-square, Mann–Whitney U and Kruskal–Wallis tests were used to compare variance according to distribution.

To calculate sample size, we used Equation (1):

(1) $$n={\left(\frac{z_{1-\alpha ∕2}\times \sqrt{p\times \left(1-p\right)}}{\delta }\right)}^{2}$$

Z_1-α/2_ is 1.96; δ represents allowable error 0.05; *p* represents sensitivity, which was determined as 0.83 in a previous study of 3-year mortality rate in post-STEMI prediabetic patients treated with PCI, in which clinical outcomes, including death, occurred in 11.2% of patients with prediabetes. Thus, we found that a minimum of >400 patients were needed to obtain concise results in this study. Taking into consideration a possible 20% drop-out rate due to several reasons, we decided to include 480 more patients. 

Spearman’s correlation coefficient was calculated to determine the correlation between variables. Plausible predictors of the combined clinical outcome were identified using univariate logistic regression and backward stepwise multivariate logistic regression. An odds ratio (OR) and 95% confidence interval (CI) were calculated for each predictor. The reliability of the predictive models was determined by receiver operating curve (ROC) analysis, with further calculation of area under the curve (AUC), its CI, sensitivity (Se), specificity (Sp) and likelihood ratio (LR) for each predictor. The Youden test was used to estimate the cut-off points for irisin and its trajectory. We compared the incremental prognostic capacity of models using a binary prediction methodology based on the estimation of integrated discrimination indices (IDI) and net reclassification improvement (NRI). The Kaplan–Meyer curve analysis was performed to elucidate a plausible benefit for clinical outcome as a function of adropin levels (≥2.15 ng/mL vs. <2.15 ng/mL). A 2-sided *p* < 0.05 was considered significant. Variables were tested using SPSS v. 23 (IBM, Armonk, New York, NY, USA) and GraphPad Prism v. 9 (GraphPad Software, San Diego, CA, USA).

## 3. Results

### 3.1. General Characteristics of the Patients

We identified 126 combined clinical endpoints, which were associated with CV death due to recurrent myocardial infarction (*n* = 62, 49.2%), acute or acutely decompensated HF (*n* = 29, 23.0%), sudden death (*n* = 21, 16.7%) and stroke (*n* = 14, 11.1%). Accordingly, we divided the entire group into two groups: with (*n* = 126) and without (*n* = 372) clinical outcomes.

The study participants had a mean age of 63 years, and 52.8% were male (Table 1). They had a mean body mass index of 26.8 ± 3.76 kg/m^2^, a mean waist circumference of 99.1 ± 5.22 cm and a mean waist-to-hip ratio of 0.92 ± 0.11 units. The comorbidity profile included dyslipidemia (85.3%), hypertension (70.8%), chronic heart failure (43.4%), atrial fibrillation (18.1%), smoking (42.4%), abdominal obesity (39.0%), left ventricular hypertrophy (93.8%) and chronic kidney disease grades 1–3 (26.3%). Among the enrolled patients, 43.7% had culprit lesion in left artery descending artery; 38.2% had culprit damage in right coronary artery; 10.8% and 7.3% exerted culprit lesion in circumflex coronary artery and left main coronary artery, respectively. Along with this, 60.4% had anterior localization of myocardial infarction. The mean values of GRACE and Gensini scores were 144 ± 37 and 32 (16–45), respectively.

All patients had stable hemodynamics at baseline, with mean LVEF of 48 (42–53), moderate enlargement of left ventricle, mean LV GLS of −14.9 (−12.1; −16.7), E/e’ of 17 ± 7 units and mean LAVI of 44 (35–55) mL/m^2^. The mean eGFR was 73 ± 15 mL/min/1.73 m^2^; the mean HOMA-IR was 7.34 ± 2.9 units; and fasting glucose was 6.10 ± 0.6 mmol/L. The mean level of NT-proBNP was 623 (172–1160) pmol/mL; the mean adropin was 2.96 (1.92–4.30) pg/mL; the mean levels of hs-TnT and hs-CRP were 0.06 (0.02–0.10) ng/mL and 4.32 (2.15–6.70) mg/L, respectively.

All individuals received optimal therapy depending on their clinical state, fasting glucose, lipid profile and comorbidities, which included antihypertensive agents (angiotensin-converting enzyme inhibitors, angiotensin-II receptor antagonists, calcium channel blockers and thiazide-like/loop diuretics), beta-blockers and ivabradine, mineralocorticoid receptor antagonists when needed, antiplatelet agents and statins. Patients with atrial fibrillation were treated with anticoagulants. We did not find significant differences between subgroups in terms of age, gender, anthropometric parameters (BMI, waist circumference, waist-to-hip ratio), the presence of CV risk factors and comorbidities, such as dyslipidemia, hypertension, chronic heart failure (including HFpEF/HFmrEF), atrial fibrillation, smoking, abdominal obesity, left ventricular hypertrophy, chronic kidney disease, GRACE score, Gensini score, systolic and diastolic blood pressure, left ventricular dimensions and ejection fraction, GLS, eGFR, HOMA-IR, fasting glucose, HbA1c, creatinine, serum uric acid, lipids, hs-CRP, NT-proBNP and hs-TnT, as well as concomitant medications apart from mineralocorticoid receptor antagonists, GLP-1 receptor agonist and SGLT2 inhibitors, which were administered frequently in free-event groups. Therefore, the patients from the event group more often had HF with mildly reduced ejection fraction and less frequently had HF with preserved ejection fraction compared with the free-event group. Yet, culprit lesions in left main and right coronary arteries were more frequently detected in the event group than in the free-event group. Of note, the mean levels of adropin were significantly higher in the free-event group compared with the event group. 

### 3.2. Spearman’s Correlation between the Levels of Biomarkers at Baseline and Other Parameters

The NT-proBNP levels were positively associated with E/e’ (r = 0.32, *p* = 0.001), LAVI (r = 0.32, *p* = 0.001), LV hypertrophy (r = 0.28, *p* = 0.001) and inversely with GLS (r = −0.37, *p* = 0.001), left ventricular ejection fraction (r = −0.36, *p* = 0.001) and eGFR (r = −0.34, *p* = 0.001) (Table 2).

Adropin levels correlated positively with left ventricular ejection fraction (r = 0.34, *p* = 0.001), GLS (r = 0.32, *p* = 0.001) and negatively with the Gensini score (r = −0.34, *p* = 0.001), LAVI (r = −0.32, *p* = 0.001), fasting plasma glucose (r = −0.32, *p* = 0.001), HOMA-IR (r = −0.29, *p* = 0.001) and HbA1c (r = −0.26, *p* = 0.001). The concentrations of hs-CRP were significantly associated with the Gensini score (r = −0.28, *p* = 0.001) and LAVI (r = −0.30, *p* = 0.001). The levels of adropin and hs-CRP demonstrated mildly positive associations with GRACE (r = −0.26, *p* = 0.001 and r = 0.28, *p* = 0.001, respectively).

### 3.3. The Reliability of the Predictive Ability of Adropin: The Results of the ROC Curve Analysis

**Figure 2:**
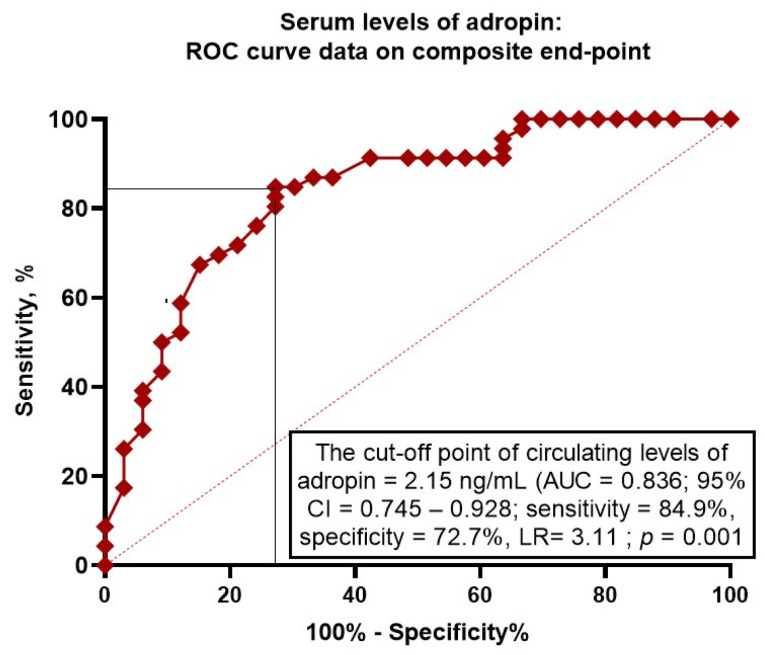
Receiver operating curve analysis for clinical events: the optimal cut-off points of adropin. Abbreviations: AUC, area under curve; CI, confidence interval; LR, likelihood ratio.

We found that serum levels of adropin < 2.15 ng/mL (area under curve [AUC] = 0.836; 95% confidence interval [CI] = 0.745–0.928; sensitivity = 84.9%; specificity = 72.7%; likelihood ratio = 3.11; *p* = 0.0001) predicted clinical outcomes (Figure 2).

### 3.4. Predictors for Clinical Events in Post-STEMI Patients with Prediabetes: Univariate and Multivariate Logistic Regression Analysis

For this analysis, we used the median value for the Gensini score, GRACE score, as well as the mean level of NT-proBNP at baseline (Table 3). The univariate logistic regression revealed that clinical events were not predicted by a GRACE score ≥ 144, serum levels of NT-proBNP ≥ 623 pmol/mL and anterior localization of myocardial infarction. On the contrary, a Gensini score ≥ 32 (OR = 1.10; *p* = 0.001), serum levels of adropin ≤ 2.15 ng/mL (OR = 1.14; *p* = 0.001), culprit lesion in left main coronary artery (OR = 1.04; *p* = 0.042), administration of SGLT2i (OR = 0.93; *p* = 0.016) and GLP-1 receptor agonist (OR = 0.95; *p* = 0.048) showed the predictive potencies for clinical outcome. The multivariate logistic regression revealed that a Gensini score ≥ 32 (OR = 1.07; *p* = 0.001), adropin ≤ 2.15 ng/mL (OR = 1.18; *p* = 0.001), use of SGLT2i (OP = 0.94; *p* = 0.010) and GLP-1 receptor agonist (OR = 0.95; *p* = 0.040) were independent predictors for clinical outcome.

### 3.5. Comparison of the Predictive Models

We compared the predictive models for clinical outcome and established that Model 2 (adropin ≤ 2.15 ng/mL) was superior to Model 1 (Gensini score ≥ 32), whereas Model 3 (administration of SGLT2i) and Model 4 (administration of GLP-1 receptor agonist) were not significantly better than the reference value of Model 1 (Table 4). Model 1 + Model 2 demonstrated better discriminative potency in comparison with Model 1.

### 3.6. Survival of the Post-STEMI Patients with Newly Diagnosed Prediabetes Depending on Serum Levels of Adropin

**Figure 3:**
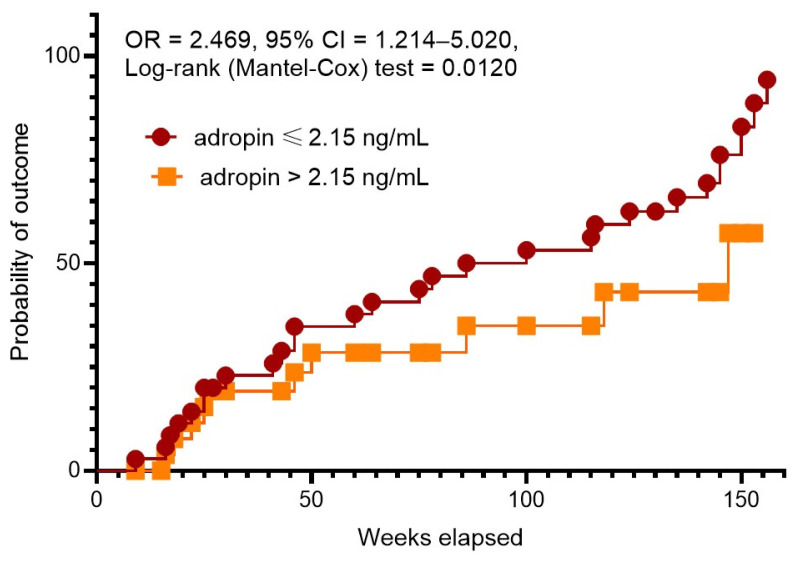
The Kaplan–Meier analysis of 3-year clinical outcomes in post-STEMI patients with newly diagnosed prediabetes depending on serum levels of adropin. Abbreviations: CI, confidence interval; OR, odds ratio.

Kaplan–Meier plots showed a significant difference between patient groups with serum levels of adropin lower than 2.15 ng/mL vs. concentrations exceeding 2.15 ng/mL (Figure 3). We found that patients with lower adropin levels had a significant benefit in clinical event occurrence compared with those with higher adropin levels (OR = 2.469; 95% CI = 1.214–5.020; log rank test = 0.0120).

## 4. Discussion

In this study, we found for the first time that among patients at moderate risk of complications after myocardial infarction and newly diagnosed prediabetes, low adropin levels were superior in their prognostic value to the traditional GRACE and Gensini risk scales. Moreover, we found that among these patients, the traditional prognostic markers, such as natriuretic peptides, C-reactive protein and troponins, did not demonstrate their discriminatory potential with respect to the combined end point, which included cardiovascular and total death. Yet, for post-STEMI patients after effective treatment with a modern strategy of complete revascularization with PCI, the risk of prediabetes as a cluster of risk factors worsening prognosis remains, and such patients do not differ from each other in most of the traditional parameters. However, a number of indicators, such as the Gensini score and adropin, retained their predictive value for clinical use. Another feature of our study is that all patients were adequately treated; yet, adropin improved the discriminative potential of Gensini for long-term events.

The prognostic significance of newly diagnosed prediabetes has not been adequately studied in patients after STEMI in contrast to diabetes mellitus, whose role in modulating complications after STEMI/PCI is fairly well established [50,51,52]. Indeed, several multicenter studies, including the post hoc analysis of the BIO-RESORT and BIONYX stent trials, have shown that prediabetes in post-STEMI patients treated with PCI resulted in a significantly higher mortality rate [53,54,55]. However, the study design was not based on newly diagnosed prediabetes after STEMI but on established diabetes before STEMI. Finally, it remains unclear whether the 3-year mortality rate for post-STEMI patients with newly diagnosed prediabetes is the same as for those who had this condition before STEMI. On the other hand, the current strategy of complete revascularization compared with the culprit-lesion-only strategy of revascularization has proven its ability to improve clinical outcomes in patients with and without diabetes mellitus, but there are a lack of data on the contribution of prediabetes to adverse clinical outcomes after complete revascularization [56]. Some clinical studies showed that prediabetes, as determined by the ADA criteria (HbA1c = 5.7%–6.5%), was either not associated with long-term adverse CV outcomes or mildly associated with clinical outcomes in patients with CAD treated with PCI [57,58], whereas ACS patients undergoing PCI, regardless of the type of revascularization (complete or culprit-lesion-only), with prediabetes were correlated with major adverse CV and cerebrovascular events (MACCEs) [59,60]. However, we established that the risk of MACCEs in STEMI patients after PCI may be independently associated with the levels of adropin.

In a previous meta-analysis, the levels of adropin were found to be decreased in overweight/obese patients vs. normal-weight individuals [61]. Aligned with this, the adropin levels were lower in patients with diabetes mellitus compared with healthy volunteers [62]. Moreover, low levels of adropin were correlated with metabolic syndrome, and perhaps this is the adaptive regulator counteracting the development of metabolic syndrome and prediabetes [63,64]. Indeed, previous studies revealed that adropin is closely related to upregulation of the endothelial nitric oxide synthase expression and peroxisome-proliferator-activated receptor-gamma in vasculature and inhibition of atherosclerosis via modulating the expression of vascular endothelial growth factor receptor 2, vascular cell adhesion molecule 1 and intercellular adhesion molecule 1, as well as a shift of the macrophage phenotype to anti-inflammatory M2 [65,66]. Moreover, regulation of adropin histone deacetylation in vivo and in vitro enhanced atherosclerosis development [67]. Although adropin was not found to trigger low-oxidative lipids’ efflux and had no significant effects on oxidized low-density lipoprotein-induced foam cell formation, it attenuated the development of plaque and its vulnerability through suppression of the inflammatory reaction [67].

In our study, we detected low levels of adropin in the enrolled patients, but we did not find an association between the levels of adropin and inflammatory biomarkers, such as hs-CRP, age and gender, whereas in the study by Butler AA et al. (2012) [64], the levels of adropin were negatively associated with age, and they were lower in females. However, there was an inverse link between the Gensini score and circulating levels of adropin. Taking into consideration these findings, we suggested that adropin seemed to be an adaptive co-regulator of metabolic homeostasis with a vasoactive component, which ensures a tissue-protective effect. In this connection, the decreased levels of adropin in post-STEMI individuals with newly diagnosed prediabetes can be considered a new pathogenetic factor contributing to microvascular inflammation, plaque instability, endothelial dysfunction, acceleration of atherosclerosis and post-conditioning [29,30,32]. Indeed, adropin is involved in downregulation of the mRNA expression of several endothelial cell markers, such as leukocyte differentiation antigen 31 and vascular endothelial cadherin, which play a pivotal role in endothelial dysfunction and plaque instability [68]. Therefore, adropin acting via transforming growth factor-beta suppresses oxidative stress and modulates lipid profiles and fibroblast activation, leading to accumulation of extracellular matrix and fibrosis [69]. Taken together, low levels of adropin, which are a result of adipose tissue inflammation and counter-regulation of inflammation, are associated with acceleration of atherosclerosis, plaque rapture, endothelial dysfunction, cardiac fibrosis and impaired vascular integrity [70]. Thus, a deficiency in adropin is probably the maladaptive mechanism that links local and systemic inflammation induced by impaired glucose homeostasis, lipid toxicity and mitochondrial dysfunction and supports functional and structural changes in coronary artery and myocardium with MACCEs [71]. 

Finally, in the study, we established that post-STEMI patients treated with PCI may demonstrate a sufficient difference in survival depending on the levels of adropin. Patients with lower levels of adropin benefited in terms of MACCEs compared with those with higher levels. These findings require a clear elucidation in a large clinical trial because the conventional risk scores, such as GRACE and Gensisni, did not reproduce their discriminative potencies for these individuals. Thus, newly diagnosed prediabetes in well-treated patients after STEMI is likely to be a sufficient risk factor for long-term survival. This is the basis for further adaptation of the currently available approach to the prediction of clinical CV outcomes in the patient population. However, clear molecular mechanisms via which adropin may be involved in tissue reparation and protection are not fully understood. 

In summary, we believe that the measurement of serum adropin levels is a simple and reasonably reliable tool for predicting the cumulative three-year risk of death. Although this is a rapid and inexpensive method of cardiovascular risk assessment that probably complements clinical assessment well and may be an effective guide for patient stratification, it needs validation in a large clinical trial setting among the full spectrum of patients with acute coronary syndrome and myocardial infarction.

### Study Limitations

The study has several limitations. First, during admission to hospital with STEMI, we did not assess the patients’ glycemia status and used findings, which were available through the discharge reports. Second, all individuals were well treated with a combination of diet and contemporary agents to reach optimal glycemia control after the determination of newly diagnosed prediabetes. Third, in the study, we did not investigate the role of SGLT2i and GLP-1 receptor agonists in the dynamics of adropin in connection with MACCE occurrence. Finally, in the study, we included patients who underwent complete and culprit-lesion-only revascularization. Overall, we believe that these limitations will not intervene with the findings’ interpretation. 

## 5. Conclusions

We established that low levels of adropin (≤2.15 ng/mL) independently predicted clinical outcomes in post-STEMI patients treated with PCI with newly detected prediabetes and improved the discriminative ability of the Gensini score for 3-year follow-up events. Future clinical studies are needed to clarify whether adropin is a promising molecule to be incorporated into conventional risk scores for the prediction of MACCEs after STEMI.

## Figures and Tables

**Table 1:** Baseline characteristics of eligible post-STEMI patients.

Variables	Entire Group (*n* = 498)	Clinical Outcomes Group (*n* = 126)	Clinical Outcomes Free Group (*n* = 372)	*p* Value
Demographics/anthropometric parameters and comorbid conditions				
Age, year	63 (51–75)	65 (52–77)	62 (50–75)	0.228
Male/female, *n* (%)	263 (52.8)/235 (47.2)	77 (61.1)/49 (38.9)	186 (50.0)/186 (50.0)	0.050
BMI, kg/m^2^	26.8 ± 3.76	26.9 ± 3.65	25.5 ± 3.90	0.720
Waist circumference, cm	99.1 ± 5.22	101.2 ± 5.70	95.3 ± 6.17	0.160
WHR, units	0.92 ± 0.11	0.92 ± 0.10	0.91 ± 0.09	0.880
Dyslipidemia, *n* (%)	425 (85.3)	113 (89.7)	312 (83.9)	0.254
Hypertension, *n* (%)	353 (70.8)	88 (69.8)	265 (71.2)	0.640
Chronic HF, *n* (%)	216 (43.4)	55 (43.7)	161 (43.3)	0.782
HFpEF, *n* (%)	43 (19.9)	11 (20.0)	32 (19.9)	0.838
HFmrEF, *n* (%)	173 (80.1)	44 (80.0)	129 (80.1)	0.840
Atrial fibrillation, *n* (%)	90 (18.1)	24 (19.0)	66 (17.7)	0.060
Smoking, *n* (%)	211 (42.4)	57 (45.2)	154 (41.4)	0.054
Abdominal obesity, *n* (%)	194 (39.0)	53 (42.1)	141 (37.9)	0.051
Left ventricular hypertrophy, *n* (%)	467 (93.8)	118 (93.7)	349 (93.8)	0.880
CKD grades 1–3, *n* (%)	131 (26.3)	37 (29.4)	94 (25.3)	0.554
Culprit lesion of coronary arteries and MI localization				
Left main coronary artery, *n* (%)	36 (7.3)	14 (11.1)	22 (6.0)	0.044
Left anterior descending artery, *n* (%)	218 (43.7)	57 (45.2)	161 (43.3)	0.722
Right coronary artery, *n* (%)	190 (38.2)	42 (33.3)	106 (28.5)	0.049
Circumflex artery, *n* (%)	54 (10.8)	13 (10.3)	41 (11.0)	0.860
Anterior localization, *n* (%)	301 (60.4)	80 (63.5)	221 (59.4)	0.050
Posterior localization, *n* (%)	197 (39.6)	46 (36.5)	151 (40.6)	0.050
Post-STEMI risk determination and severity of atherosclerosis				
GRACE score	144 ± 37	148 ± 31	141 ± 41	0.620
Gensini score	32 (16–45)	35 (18–46)	31 (14–43)	0.144
Hemodynamic features				
Systolic BP, mm Hg	141 ± 8	140 ± 8	139 ± 9	0.664
Diastolic BP, mm Hg	84 ± 7	87 ± 6	81 ± 8	0.612
LVEDV, mL	159 (138–167)	162 (144–172)	156 (137–166)	0.474
LVESV, mL	83 (74–94)	89 (82–100)	81 (73–92)	0.680
LVEF, %	48 (42–53)	45 (41–51)	47 (43–52)	0.854
LVMMI, g/m^2^	138 ± 11	140 ± 13	136 ± 10	0.442
LAVI, mL/m^2^	44 (35–55)	45 (36–56)	42 (34–53)	0.124
E/e’, unit	17 ± 7	18 ± 6	15 ± 5	0.412
GLS, %	−14.9 (−12.1; −16.7)	−15.1 (−13.2; −16.9)	−14.2 (−11.9; −16.1)	0.354
Biomarkers				
eGFR, mL/min/1.73 m^2^	73 ± 15	66 ± 13	81 ± 15	0.126
HOMA-IR, units	7.34 ± 2.9	7.35 ± 2.8	7.22 ± 2.5	0.740
Fasting glucose, mmol/L	6.10 ± 0.6	6.11 ± 0.5	6.08 ± 0.7	0.880
HbA1c, %	5.92 ± 0.30	5.91 ± 0.24	5.90 ± 0.29	0.845
Creatinine, µmol/L	100.8 ± 15.1	116.9 ± 14.7	99.1 ± 15.3	0.412
SUA, mcmol/L	321 ± 109	361 ± 111	303 ± 78	0.662
Total cholesterol, mmol/L	4.92 ± 1.15	5.05 ± 1.13	4.77 ± 1.18	0.486
HDL-C, mmol/L	0.93 ± 0.60	0.94 ± 0.43	0.93 ± 0.60	0.752
LDL-C, mmol/L	3.38± 0.46	3.47± 0.25	3.24 ± 0.22	0.650
Triglycerides, mmol/L	1.35 ± 0.31	1.40 ± 0.27	1.33 ± 0.29	0.774
hs-CRP, mg/L	4.32 (2.15–6.70)	4.60 (2.15–7.30)	4.12 (2.10–6.56)	0.120
Adropin, pg/mL	2.96 (1.92–4.30)	2.14 (1.80–2.83)	3.64 (2.70–5.58)	0.001
NT-proBNP, pmol/mL	623 (172–1160)	691 (210–1250)	611 (170–1088)	0.420
hs-TnT, ng/mL	0.06 (0.02–0.10)	0.06 (0.01–0.08)	0.05 (0.01–0.10)	0.742
Concomitant medications				
ACE inhibitors, *n* (%)	316 (63.5)	80 (63.5)	236 (63.4)	0.892
Angiotensin-II receptor blockers, *n* (%)	78 (15.7)	21 (16.7)	57 (15.3)	0.524
Beta-blockers, *n* (%)	456 (91.6)	115 (91.3)	341 (91.7)	0.832
Ivabradine, *n* (%)	82 (16.5)	21 (16.7)	61 (16.4)	0.840
Calcium channel blockers, *n* (%)	61 (12.2)	16 (12.7)	45 (12.1)	0.828
Thiazide-like diuretics, *n* (%)	43 (8.6)	11 (8.7)	32 (8.6)	0.866
Loop diuretics, *n* (%)	216 (43.4)	55 (43.7)	161 (43.3)	0.782
MRA, *n* (%)	173 (80.1)	39 (70.9)	134 (83.2)	0.040
Antiplatelet agents, *n* (%)	429 (86.4)	112 (88.9)	317 (85.2)	0.820
Anticoagulants, *n* (%)	90 (18.1)	24 (19.0)	66 (17.7)	0.060
Metformin, *n* (%)	116 (23.3)	28 (22.2)	88 (23.7)	0.760
GLP-1 receptor agonist, *n* (%)	95 (19.1)	18 (14.3)	77 (20.7)	0.044
SGLT2 inhibitors, *n* (%)	297 (59.6)	59 (43.7)	238 (64.0)	0.010
Statins, *n* (%)	498 (100.0)	126 (100.0)	372 (100.0)	1.000

Notes: Variables are given as M ± SD and Me (25–75% IQR). Chi-square test was used to compare categorical variables. The Mann–Whitney U-test and Kruskal–Wallis test were used to compare continuous variables between cohorts. Abbreviations: BMI, body mass index; CKD, chronic kidney disease; eGFR, estimated glomerular filtration rate; E/e’, early diastolic blood filling to longitudinal strain ratio; GRACE, Global Registry of Acute Coronary Events; GLS, global longitudinal strain; GLP-1, glucagon-like peptide-1; HbA1c, glycated hemoglobin; HDL-C, high-density lipoprotein cholesterol; HFpEF, heart failure with preserved ejection fraction; HFmrEF, heart failure with mildly reduced ejection fraction; HOMA-IR, homeostatic assessment model of insulin resistance; hs-CRP, high-sensitivity C-reactive protein; LAVI, left atrial volume index; LDL-C, low-density lipoprotein cholesterol; LVEDV, left ventricular end-diastolic volume; LVESV, left ventricular end-systolic volume; LVEF, left ventricular ejection fraction; LVMMI, left ventricle myocardial mass index; NT-proBNP, N-terminal natriuretic pro-peptide; MRA, mineralocorticoid receptor antagonists; SGLT2, sodium-glucose co-transporter-2; SUA, serum uric acid; WHR, waist-to-hip ratio.

**Table 2:** Spearman’s correlation coefficients between the levels of biomarkers and other parameters.

Variables	NT-proBNP		Adropin		hs-CRP	
	r Coefficient	*p* Value	r Coefficient	*p* Value	r Coefficient	*p* Value
LVH	0.28	0.001	−0.160	0.122	0.20	0.054
LAVI	0.32	0.001	−0.32	0.001	0.30	0.001
E/e’	0.32	0.001	−0.19	0.062	0.11	0.246
GLS	−0.37	0.001	0.32	0.001	−0.07	0.366
LVEF	−0.36	0.001	0.34	0.001	−0.041	0.622
eGFR	−0.34	0.001	−0.16	0.060	−0.13	0.255
Gensini score	0.10	0.058	−0.34	0.001	−0.28	0.001
GRACE score	0.13	0.054	0.26	0.001	0.28	0.001
Fasting plasma glucose	0.063	0.774	−0.32	0.001	0.009	0.811
HOMA-IR	0.140	0.226	−0.29	0.001	0.061	0.770
HbA1c	0.080	0.516	−0.26	0.001	0.104	0.623

Abbreviations: E/e’, early diastolic blood filling to longitudinal strain ratio; GLS, global longitudinal strain; HOMA-IR, homeostatic assessment model of insulin resistance; GRACE, Global Registry of Acute Coronary Events; LVEF, left ventricular ejection fraction; LVH, left ventricular hypertrophy; LAVI, left atrial volume index; eGFR, estimated glomerular filtration rate; NT-proBNP, N-terminal natriuretic pro-peptide; hs-CRP, high-sensitivity C-reactive protein.

**Table 3:** Predictors for clinical events: the results of logistic regression.

Variables	Dependent Variables: Clinical Events					
	Univariate Log Regression			Multivariate Log Regression		
	OR	95% CI	*p* Value	OR	95% CI	*p* Value
GRACE score ≥ 144 vs. <144	1.06	1.00–1.12	0.064	-		
Gensini score ≥ 32 vs. <32	1.10	1.02–2.12	0.001	1.07	1.04–2.10	0.001
NT-proBNP at baseline ≥ 623 pmol/mL vs. <690 pmol/mL	1.05	0.98–1.15	0.810	-		
Adropin ≤ 2.15 ng/mL vs. >2.15 ng/mL	1.14	1.04–1.52	0.001	1.18	1.03–1.36	0.001
Culprit lesion in left main coronary artery	1.04	1.02–1.08	0.042	1.04	1.00–1.09	0.052
Localization of MI, anterior vs. posterior	1.03	1.00–1.07	0.168	-		
Administration of SGLT2i vs. unused SGLT2i	0.93	0.89–0.98	0.016	0.94	0.89–0.97	0.010
Administration of GLP-1 receptor agonist vs. unused GLP-1 receptor agonist	0.95	0.92–0.99	0.048	0.95	0.90–0.99	0.040

Abbreviations: OR, odds ratio; CI, confidence interval; GLP-1, glucagon-like peptide-1; MI, myocardial infarction; SGLT2i, sodium-glucose co-transporter-2 inhibitor; NT-proBNP, N-terminal natriuretic pro-peptide.

**Table 4:** The comparisons of predictive models for clinical outcomes.

Predictive Models	Dependent Variable: Clinical Outcome					
	AUC		NRI		IDI	
	M (95% CI)	*p* Value	M (95% CI)	*p* Value	M (95% CI)	*p* Value
Model 1 (Gensini score ≥ 32)	0.802 (0.732–0.877)	-	Reference	-	Reference	-
Model 2 (Adropin ≤ 2.15 ng/mL)	0.836 (0.745–0.928)	0.012	0.45 (0.37–0.54)	0.010	0.50 (0.44–0.57)	0.010
Model 3 (Administration of SGLT2i)	0.815 (0.738–0.886)	0.724	0.14 (0.11–0.19)	0.266	0.16 (0.12–0.21)	0.442
Model 4 (Administration of GLP-1 receptor agonist)	0.811 (0.740–0.875)	0.760	0.13 (0.10–0.16)	0.460	0.15 (0.10–0.21)	0.514
Model 1 + Model 2	0.936 (0.885–0.987)	0.040	0.52 (0.46–0.61)	0.044	0.54 (0.41–0.66)	0.020

Abbreviations: AUC, area under curve; CI, confidence interval; IDI, integrated discrimination indices; NRI, net reclassification improvement. Note: *p* value indicates a significant difference in terms of Model 1.

## Data Availability

The data presented in this study are available on request from the corresponding author due to privacy restrictions.
